# A Genetic Porcine Model of Cancer

**DOI:** 10.1371/journal.pone.0128864

**Published:** 2015-07-01

**Authors:** Lawrence B. Schook, Tiago V. Collares, Wenping Hu, Ying Liang, Fernanda M. Rodrigues, Laurie A. Rund, Kyle M. Schachtschneider, Fabiana K. Seixas, Kuldeep Singh, Kevin D. Wells, Eric M. Walters, Randall S. Prather, Christopher M. Counter

**Affiliations:** 1 Department of Animal Sciences, University of Illinois, Champaign-Urbana, Illinois, United States of America; 2 Department of Bioengineering, University of Illinois, Champaign-Urbana, Illinois, United States of America; 3 Department of Surgery, University of Illinois, Chicago, Illinois, United States of America; 4 Technology Development Center, Postgraduate Program in Biotechnology, Federal University of Pelotas, Pelotas, Rio Grande do Sul, Brazil; 5 State Key Laboratory of AgroBiotechnology, China Agricultural University, Beijing, China; 6 Department of Internal Medicine, University of Kentucky, Lexington, Kentucky, United States of America; 7 Veterinary Diagnostic Laboratory, University of Illinois, Champaign-Urbana, Illinois, United States of America; 8 Division of Animal Science, National Swine Research and Resource Center, University of Missouri, Columbia, Missouri, United States of America; 9 Department of Pharmacology & Cancer Biology, Duke University Medical Center, Durham, North Carolina, United States of America; 10 Department of Radiation Oncology, Duke University Medical Center, Durham, North Carolina, United States of America; Friedrich-Loeffler-Institute, GERMANY

## Abstract

The large size of the pig and its similarity in anatomy, physiology, metabolism, and genetics to humans make it an ideal platform to develop a genetically defined, large animal model of cancer. To this end, we created a transgenic “oncopig” line encoding Cre recombinase inducible porcine transgenes encoding KRAS^G12D^ and TP53^R167H^, which represent a commonly mutated oncogene and tumor suppressor in human cancers, respectively. Treatment of cells derived from these oncopigs with the adenovirus encoding Cre (AdCre) led to KRAS^G12D^ and TP53^R167H^ expression, which rendered the cells transformed in culture and tumorigenic when engrafted into immunocompromised mice. Finally, injection of AdCre directly into these oncopigs led to the rapid and reproducible tumor development of mesenchymal origin. Transgenic animals receiving AdGFP (green fluorescent protein) did not have any tumor mass formation or altered histopathology. This oncopig line could thus serve as a genetically malleable model for potentially a wide spectrum of cancers, while controlling for temporal or spatial genesis, which should prove invaluable to studies previously hampered by the lack of a large animal model of cancer.

## Introduction

A large animal model of cancer would be beneficial in settings requiring size, anatomy, metabolism, or genetics reflective of humans, such as for studies of noninvasive image-guided technologies, radiation oncology, drug metabolism, surgical training, technology development (e.g., early detection screening), to name but just a few [1,2]. In this regard, the pig is an ideal large animal platform to develop such a model. These are large mammals with similar anatomy to humans [1,2]. Both their basal metabolic rate and their xenosensor pregnane X receptor that regulates *CYP3A* expression, which is responsible for the metabolism of half of all prescription drugs [3], are also very similar to humans [4,5]. Like humans, pigs also require multiple genetic changes to develop cancer [6].

To develop a porcine model of cancer, we chose to recapitulate those mutations most commonly found in human cancer. Previously, we had demonstrated that human mutations when provided in gene expression studies using autologous porcine fibroblasts resulted in oncogenes molecularly similar to that observed in humans and with similar pathology [6]. In this study, we have targeted the *RAS* gene which is mutated in one quarter of all human cancers, with the *KRAS* isoform being the most commonly mutated [7] yielding a constitutively active, oncogenic protein [7] that experimentally induces cancer in mice [8] and human cells [9], in addition to underlying a number of hereditary cancer syndromes [10]. The gene *TP53* is mutated in a third of human cancers [11] to silence this tumor suppressive pathway, which similarly is known to promote cancer in both mouse [12] and porcine [13] genetic models as well as in human cells [9], and is associated with the cancer predisposition Li-Fraumeni Syndrome [14]. Mutations in these two genes occur in concert in human cancers (COSMIC) [15]. Moreover, mice genetically engineered to undergo recombination to convert their wild-type *Kras* and *TP53* alleles to oncogenic and dominant-negative or null versions, respectively, rapidly develop aggressive cancers at the sites of recombination [16,17]. With regards to pigs, both oncogenic *Kras* and dominant-negative *p53* help promote the tumorigenic conversion of normal porcine cells to a tumorigenic state [6], and pigs engineered with a mutant TP53 allele are prone to develop lymphomas and osteogenic tumors [13]. Most recently, a conditionally activated oncogenic KRAS mutation in pigs has been reported [18]. As such, we chose to engineer pigs with inducible expression of these commonly mutated and potent oncogene and tumor suppressor genes.

## Results

### Creation of oncopigs encoding inducible oncogenic KRAS and dominant-negative TP53

**Fig 1:**
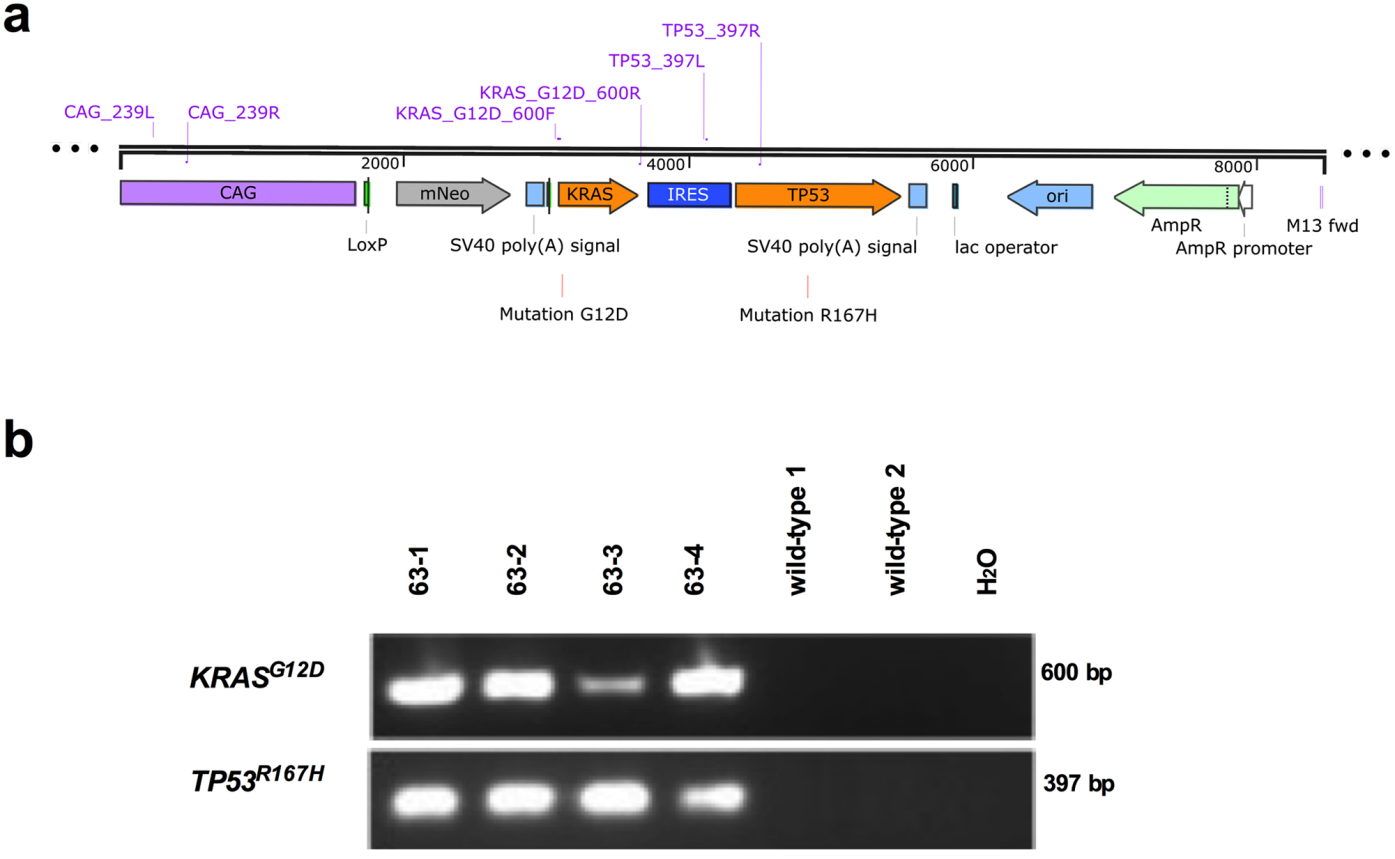
The Cre-inducible vector encoding *KRAS* ^*G12D*^ and *TP53* ^*R167H*^. (*a*) Schematic diagram of the vector encoding Cre-inducible *KRAS* ^*G12D*^ and *TP53* ^*R167H*^. (*b*) PCR analysis for the presence of *KRAS* ^*G12D*^ and *TP53* ^*R167H*^ in genomic DNA isolated from the indicated cloned offspring.

To create an inducible porcine model of cancer, which we term “oncopig”, we first cloned the porcine *KRAS* and *TP53* cDNAs from the Duroc pig (2–14, TJ Tabasco) that was used to sequence the pig genome [19]. Site-directed mutagenesis was then used to introduce the oncogenic G12D mutation into the porcine *KRAS* cDNA. This mutation was chosen as an aspartic acid substitution accounts for over a third of the mutations at the G12 position in human cancers [7] and introducing this mutation into the endogenous murine *Kras* gene promotes tumorigenesis [8,20]. Similarly, the R167H mutation was chosen as its human equivalent (R175H) is commonly found in human cancers [11] as well as the cancer predisposition Li-Fraumeni Syndrome [14], and when introduced into the endogenous murine [12] or porcine [13] *TP53* gene, induces tumors. These two cDNAs were then introduced into a Cre-inducible vector, yielding an expression construct containing the CAG promoter, followed by the aforementioned LSL sequence, *KRAS* ^*G12D*^, an IRES sequence to allow for bicistronic expression, *TP53* ^*R167H*^ and a poly A sequence (Fig 1a). This design allows for co-expression of both *KRAS* ^*G12D*^ and *TP53* ^*R167H*^ in ostensibly any cells of the pig by transient infection with AdCre, which in principle should allow induction of a broad range of cancers in specific tissue sites and at any chosen time.

Normal porcine embryonic fibroblasts [21] were transfected with the above plasmid and stably transfected cell clones selected in G418-supplemented medium. Genomic DNA from the cell colonies was used to verify the presence of both transgenes (*KRAS* ^*G12D*^ and *TP53* ^*R167H*^) by PCR assayed using primers specific for these transcripts, and were subsequently expanded for the source of nuclei for nuclear transfer. Nuclei from these cells were then isolated and transferred to enucleated porcine oocytes and embryogenesis activated, a process termed somatic cell nuclear transfer (SCNT) [21]. A total of >100 such embryos were transferred to a surrogate sow, which yielded four male oncopig offspring (63–1, 63–2, 63–3, 63–4). PCR analysis of isolated genomic DNA using primers specific for these transgenes confirmed stable integration of the *KRAS* ^*G12D*^ and *TP53* ^*R167H*^ cDNAs (Fig 1b).

### Cre activation of the *KRAS* ^*G12D*^ and *TP53R167H* transgenes in fibroblasts derived from the oncopigs is transforming

**Fig 2:**
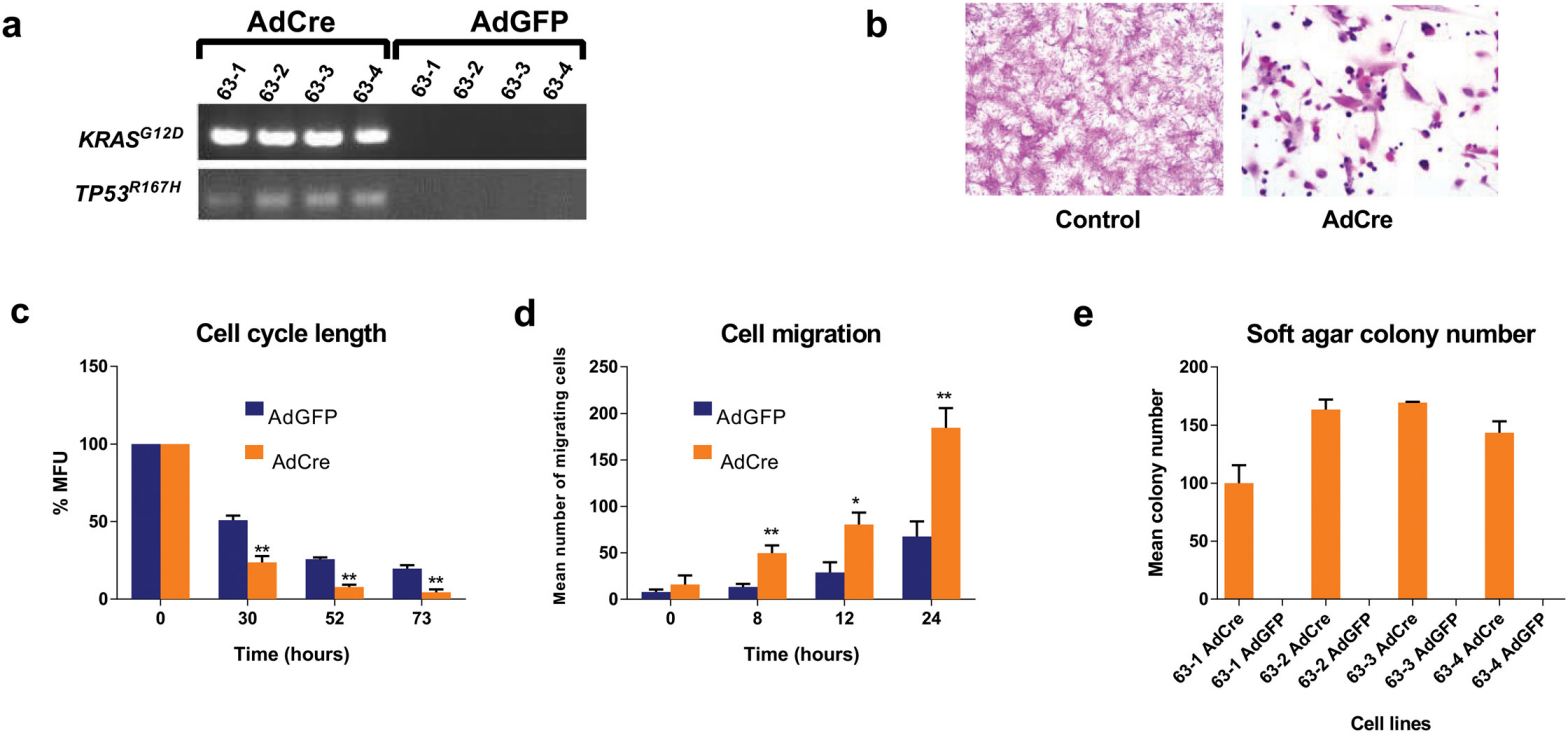
Inducible expression of *KRAS* ^*G12D*^ and *TP53* ^*R167H*^ is transforming and tumorigenic. (*a*) RT-PCR analysis of *KRAS* ^*G12D*^ and *TP53* ^*R167H*^ mRNA expression in the fibroblast cell lines from each of the 4 transgenic clones treated with AdCre or AdGFP. (*b*) Comparison of cell morphology between AdCre and untreated control cells in culture, stained with H&E. (*c*) Normalized MFU measured by FACS at time points following Carboxyfluorescein succinimidyl ester (CFSE) dye loading of cells. (*d*) Graphical analysis of the mean number of migrating cells from triplicate plating of each of the 4 cell lines. (*e*) Graphical analysis of the mean number of colonies growing in soft agar for each cell line from triplicate plating. (*c-e*: all data points are the mean of the 4 cell lines derived from pigs 63–1, 63–2, 63–3, 63–4; error bars = SD; *p-value ≤ 0.05; **p-value ≤ 0.01).

To assess whether the *KRAS* ^*G12D*^ and *TP53* ^*R167H*^ transgenes could be activated to promote tumorigenesis, skin biopsies were isolated from the aforementioned four oncopig offspring, and used to establish fibroblast lines. These individual cell lines from transgenic oncopigs were infected with either adenovirus encoding the marker green fluorescence protein (AdGFP) or adenovirus encoding Cre recombinase (AdCre), and the resultant matched pairs of infected cultures were assayed for transformed and tumorigenic phenotypes. As expected, only the AdCre exposed cells exhibited detectable levels of *KRAS* ^*G12D*^ and *TP53* ^*R167H*^ mRNA, as assessed by RT-PCR (Fig 2a). Additionally, there was a clear difference in the morphology of the AdCre cells compared to the control AdGFP cells. Specifically, the former lost the spindle morphology characteristic of either the same cells prior to AdCre infection or when infected with the AdGFP control virus, and instead were smaller, rounder much more loosely attached to the plate and would spontaneously form foci (Fig 2*B*). This difference foreshadowed other transformed phenotypes. Specifically, FACS analysis revealed mean cell cycle length of all four lines was shortened from 22 hours in the AdGFP control population to 13 hours in the AdCre population (Fig 2c). AdCre cells also exhibited a mean 2.8 fold increase in cell migration of all four lines throughout the entire time course of observation (Fig 2d), as assessed by a scratch assay, and produced an average of 143 colonies when plated in soft agar compared to no colonies detected in the control AdGFP cells (Fig 2e). We concluded that fibroblast lines developed from independently derived *KRAS* ^*G12D*^/*TP53* ^*R167H*^ oncopig clones were induced to be transformed upon activation of the expression of these transgenes by Cre recombinase. AdGFP treated transgenic cells maintained phenotypes similar to those observed for non-transgenic cell lines.

### Cre activation of the *KRAS* ^*G12D*^ and *TP53* ^*R167H*^ transgenes in fibroblasts derived from the oncopigs is tumorigenic

**Fig 3:**
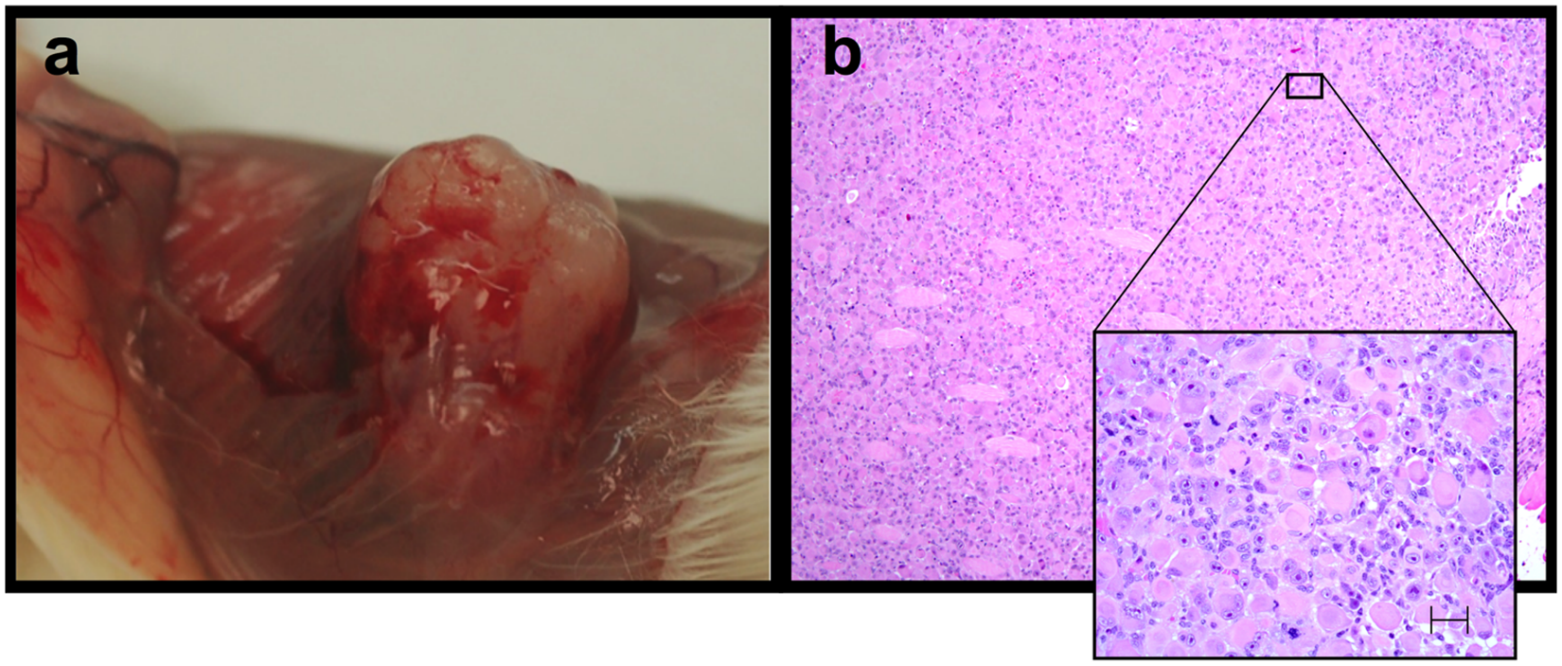
Tumors arose from each AdCre treated cell line injected into immune compromised mice. Similar tumors (a) developed from all AdCre cell lines and no tumors developed from AdGFP cell injections; Histological analysis (b) revealed the tumors to be densely cellular non encapsulated and infiltrative neoplasm. 10x and insert 40X H&E Stain.

Encouraged by the development of transformed phenotypes, we next assessed whether AdCre could induce the above fibroblasts to grow in a tumorigenic fashion. Thus, AdCre treated cells derived from four independently-derived transgenic oncopigs were injected into immuno-compromised female mice and the site of injection monitored for the development of tumors. Palpable tumors reached 2000 mm^3^ (the maximal allowed size) between 32 and 63 days post-injection—with complete penetrance days (Table 1). In contrast, no tumors were detected at the sites injected with cells from the transgenic oncopig cells treated with AdGFP over a 130-day period of observation (Table 1). We conclude that fibroblasts isolated from independent *KRAS* ^*G12D*^/*TP53* ^*R167H*^ oncopigs were induced to be tumorigenic upon activation of the expression of these transgenes by Cre recombinase. The tumor masses resulting from AdCre activation of cells from transgenic oncopigs (Fig 3) revealed under both high and low magnification evidence of solid tumor (Fig 3b) and were not the result of inflammation or fluid accumulation.

**Table 1:** Induction of Tumors Following Injection of AdCre or AdGFP treated Oncopig Transgenic Cells into Immunocompromised Mice (Days to 2000 mm^3^).

Mouse	Oncopig Transgenic Cell Line							
	63–1		63–2		63–3		63–4	
	AdCRE^1^	AdGFP	AdCRE^1^	AdGFP	AdCRE^1^	AdGFP	AdCRE^1^	AdGFP
1	9	NPG^2^	9	NPG	59	NPG	59	NPG
2	32	NPG	13	NPG	115	NPG	58	NPG
3	55	NPG	76	NPG	59	NPG	65	NPG
4	ND	ND	ND	ND	20	NPG	65	NPG
**Mean**	32		32		63		60	

^1^ For each cell line 5x10^6^ cells were mixed with Matrigel (BD Biosciences, San Diego, CA, USA) and injected subcutaneously into the flanks (left flank, AdCre and right flank, AdGFP) of 3 or 4 severe combined immunodeficient female mice (NOD.CB17-Prkdcscid/ JAX, Bar Harbor, ME).

^2^ No palpable growth (NPG) was identified for any of the AdGFP cell lines.

The tumor tissue included nonencapsulated, densely cellular, and locally infiltrative neoplasm. Neoplastic cells were arranged in sheets and bundles supported by very fine fibrovascular stroma. Individual neoplastic cells were round to spindeloid with mild to abundant fibrillary to eosinophilic cytoplasm. Nuclei were single to multiple, with round to oval shape and contain single prominent nucleolus and scattered chromatin. Neoplastic cells exhibited moderate to marked anisocytosis and anisokaryosis. Mitotic figures range from 3 to 8 per 10 high power fields (hpf). Areas of necrosis were scattered within the tumor. Neoplastic cells had invaded surrounding tissues including skeletal muscle, epidermis, kidney and ovary. Small numbers of lymphocytes and rare neutrophils were scattered within the tumors.

### Intramuscular injection of AdCre in oncopigs reproducibly induces tumors at the site of injection

Given the above observations, we next tested whether exposure of AdCre *in vivo* would induce tumors at the site of injection in transgenic oncopigs. To this end, pig 63–3 was used to produce a cohort of transgenic littermate oncopigs for this analysis. Pig 63–3 was selected following DNA sequence analysis indicating that the transgene construct was inserted at a single location within intron 4 of the porcine CCDC-129 gene (coiled-coil domain containing 129) located on SSC18. Furthermore, the insertion was oriented in a 3’ to 5’ direction relative to the chromosome and there was no evidence of a concactomer. Thus, the oncogene construct (Fig 1a) from boar 63–3 would be inherited as a single autosomal gene.

**Fig 4:**
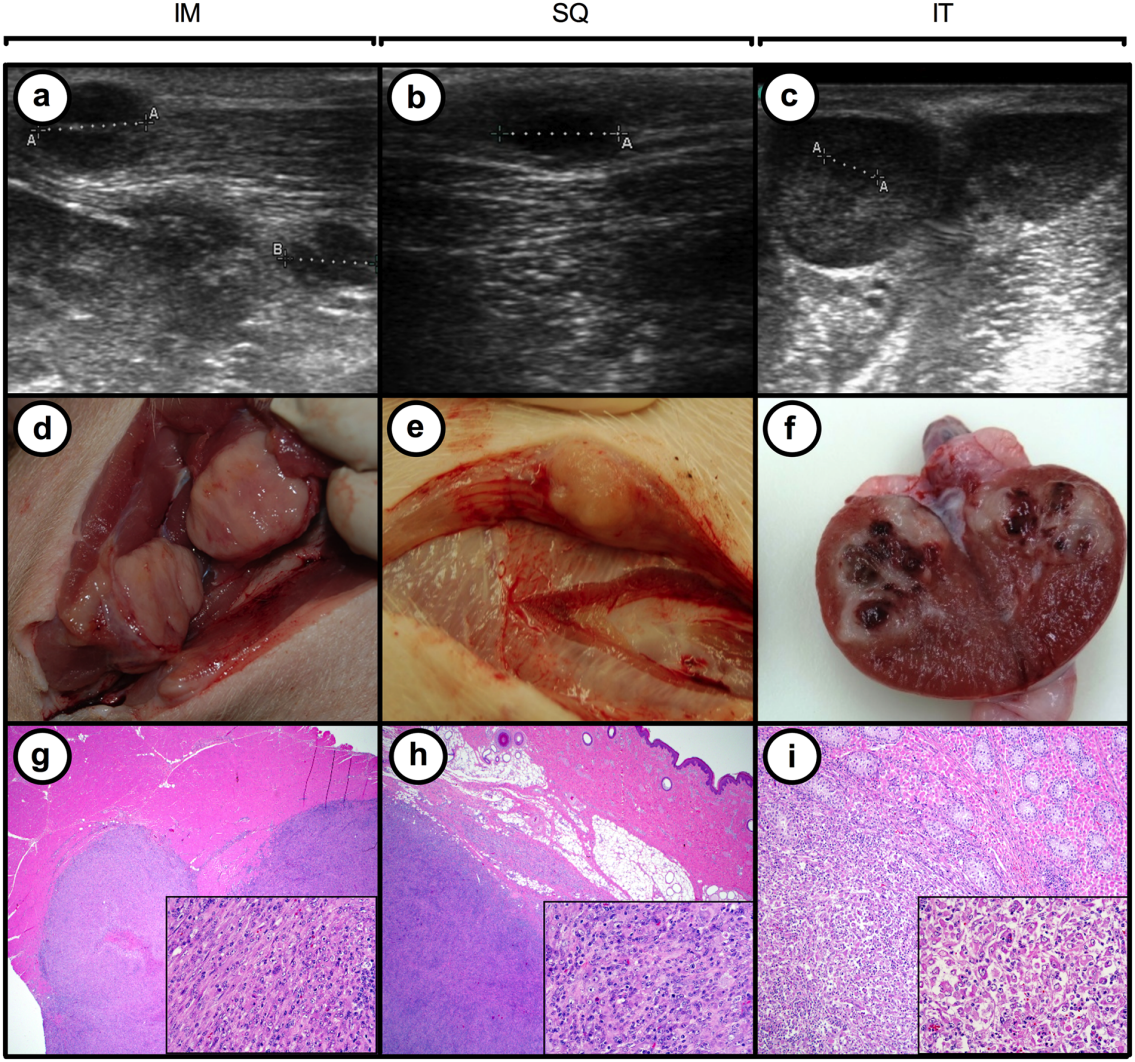
Inducible expression of *KRAS* ^*G12D*^ and *TP53* ^*R167H*^ is tumorigenic in transgenic pigs. (*a–c*) Ultrasound images of tumors developing 10 days after, and (*d–f*) images of these tumors at necropsy 20 days after intramuscular (IM, Pig 1), subcutaneous (SQ, Pig 3), or intra-testicular (IT, Pig 1) injection of AdCre in transgenic oncopigs containing a Cre-inducible vector encoding *KRAS* ^*G12D*^ and *TP53* ^*R167H*^; (g–i) H&E stained sections show the tumor and adjacent normal tissue (2x) with an inserted high magnification photo (20x).

**Fig 5:**
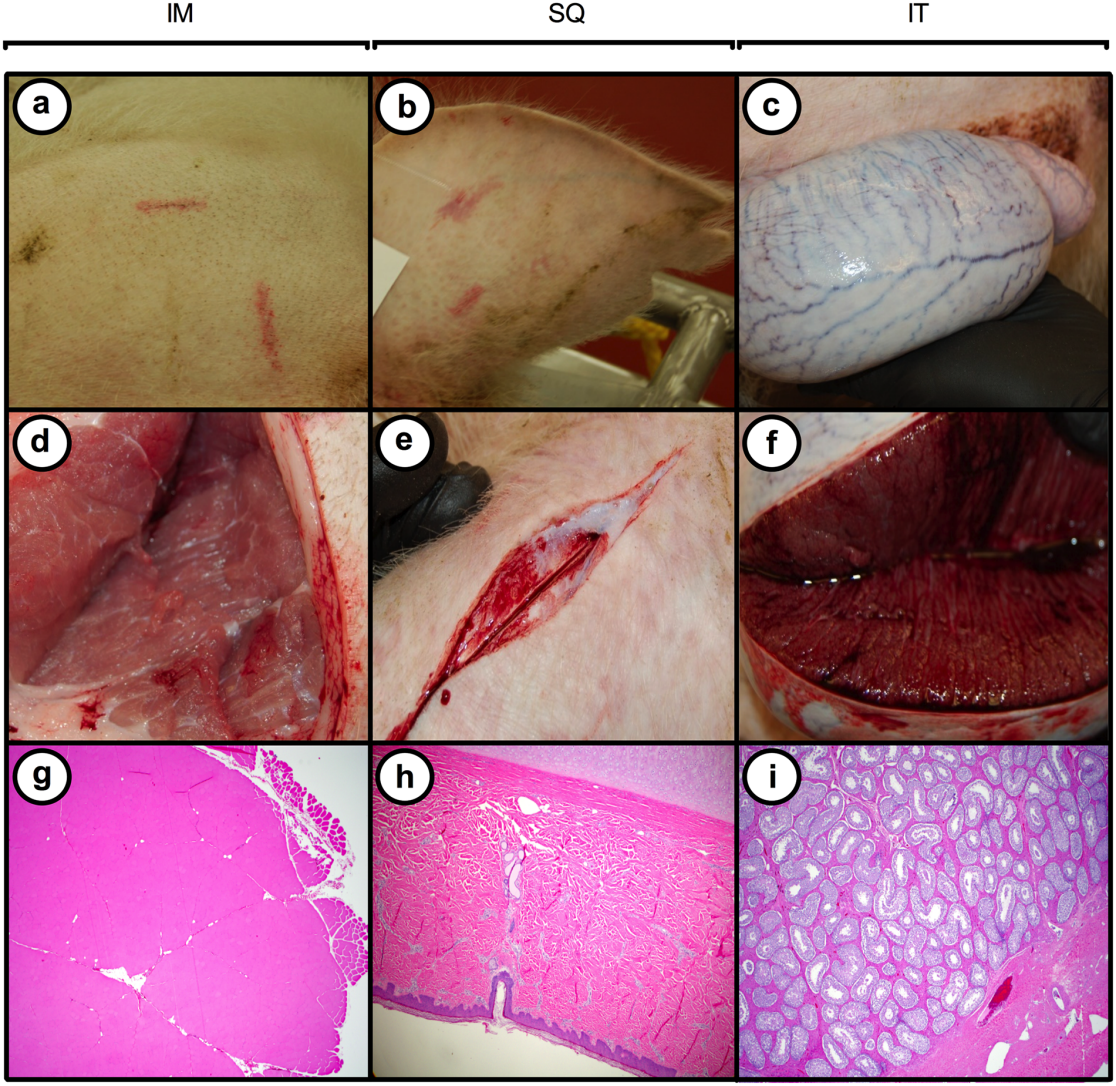
Injection of AdGFP into transgenic pigs did not induce tumors. The lack of any detectable changes to the injection site on d20 post- injection is shown at the surface (a–c); in underlying tissue (d–f); or upon histological examination (g–i).

The first of these, oncopig-1, was administered AdCre intramuscularly into the left hind leg. A tumor mass was detected by day 10 post-injection, which was clearly visible by ultrasound (Table 2 and Fig 4*a* and 4*d*). Not only was this tumor rapidly established, but it also grew quickly, reaching a volume of 8 cm^2^ within 20 days. Pathologic analysis of H&E stained sections of the tumor revealed this tumor to be of mesenchymal origin (Fig 4g and Table 2). Specifically, the tumor was microscopically characterized as a densely cellular, non-encapsulated and locally infiltrative neoplasm. The cells are arranged in bundles, streams and small sheets of supported by a fibrous stroma. Individual neoplastic cells were pleomorphic round to oval to polygonal with single to multiple nuclei. Areas of necrosis and chronic inflammation were scattered within the tumor (Fig 4*g*). Neoplastic cells were stained positively with vimentin and smooth muscle markers (muscle specific actin and smooth muscle actin). This was a reproducible result, as intramuscular injection of AdCre into the other rear leg similarly yielded a tumor of mesenchymal origin at the site of injection (Table 2). Moreover, tumorigenesis was induced in other littermate offspring, indicating this was not unique to a single animal tested. Specifically, AdCre was injected intramuscularly into two legs and the neck of oncopig-2, resulting in tumor masses at all three injection sites, with pathological analysis of H&E stained sections again supporting a mesenchymal tumor diagnosis (Table 2). Isolation of RNA from the resulting tumor masses was used to monitor the expression of mutant transgenic transcripts (Table 2). Both *KRAS* ^*G12D*^ and *TP53* ^*R167H*^ transgenes were expressed in tumors excised at autopsy. The expression of mutant over wild type transcripts was elevated in each of the sites monitored. Control non-transgenic animals exposed to AdCre and transgenic pigs exposed to AdGFP did not reveal any tumor masses nor pathological changes (Fig 5).

**Table 2:** Summary of porcine tumor growth in vivo.

Animal^a^	Injection Site^b^	Dose^c^	Ultrasound Diameter^d^	Tumor Size^e^	Kras^G12D^ Expression^f^	p53^R167H^ Expression^f^	Pathology
Pig 1	IM (Right Leg)	2x109	1.25	3 x 1.5	15.83	16.34	Mesenchymal Tumor
Pig 1	IM (Left Leg)	2x109	1.12	4 x 2	12.55	8.28	Mesenchymal Tumor
Pig 1	IT (Right Testis)	2x109	0.63	2 x 1.5			Mesenchymal Tumor
Pig 2	IM (Right Leg)	1x109	1.72	1.3 x 0.7	44.01	20.71	Mesenchymal Tumor
Pig 2	IM (Left Leg)	1x109	0.91	3.3 x 1	94.55	6.60	Mesenchymal Tumor
Pig 2	IM (Neck)	1x109	0.6	1 x 1.2			Mesenchymal Tumor
Pig 3	SQ (Right Ear)	1x109	0.62	0.7 x 0.6			Mesenchymal Tumor
Pig 3	SQ (Right Neck)	1x109	1.02	2 x 0.8			Mesenchymal Tumor
Pig 3	SQ (Right Abdomen)	1x109	0.93	1 x 2			Mesenchymal Tumor

^a^ Offspring sired by clone 63–3

^b^IM, intramuscular; SQ, subcutaneous; IT, intra-testicular

^c^Concentration of 2x10^9^ pfu AdCre/ml

^d^Size 10 days post-injection (cm)

^e^Size 20 days post-injection (cm)

^f^Ratio of normalized RNAseq levels between AdCre induced tumor tissue RNA/untreated transgenic muscle tissue RNA

### Different tumor types can be induced in the oncopig based upon the injection site of AdCre

To explore whether injection of AdCre in other tissues could lead to tumors, a testis of oncopig-1 was injected with AdCre. Again, a palpable mass was detected 10 days post-injection that rapidly developed into a tumor (Fig 4*c*, 4*f*, 4*i* and Table 2). Histopathology of this tumor revealed a poorly differentiated tumor likely of sex cord stromal origin. Similarly, the ear, abdomen and neck of oncopig-3 were injected subcutaneously with AdCre, which again led to tumor masses at all three sites with the pathology of sarcoma with regional smooth muscle differentiation (leiomyosarcoma) (Fig 4*b*, 4*e*, 4*h* and Table 2). Thus, the administration of AdCre reproducibly induced tumorigenesis in transgenic oncopigs at each injection site. The administration of AdGFP into additional oncopigs did not result in the generation of tumor mass or pathology (Fig 5).

## Discussion

The pig has many attributes that make it an ideal platform to develop a genetically defined, large animal model of cancer. We now report the creation of a transgenic oncopig line encoding a Cre recombinase inducible transgene encoding *KRAS* ^*G12D*^ and *TP53* ^*R167H*^, a commonly mutated oncogene [7] and tumor suppressor [11], respectively, in human cancers. This study is an extension on our previous work [6] where we demonstrated that mutations in genes identified in human studies when inserted into porcine genes did result in oncogenesis and pathology that replicated tumors observed clinically.

In validating the porcine cancer animal model it is imperative that the resulting tumor masses are validated with what is observed in human medicine. This need has been clearly articulated by Cardiff [22–24] where the neoplastic progression using the mouse for breast cancer using human mutations failed to replicate tumors observed in the clinic. Thus, this study focused on demonstrating that genetically engineered porcine tumors result in histopathological phenotypes that span from signature phenotypes to mimicking human cancers [24].

Thus, the porcine model was validated by the demonstration that derived fibroblasts from transgenic oncopigs treated with AdCre demonstrated activation of the transgene, leading to all the *in vitro* hallmarks of tumors, including a transformed cell morphology, increased proliferation and migration, and the ability to growth in an anchorage-independent fashion. In agreement, these cells were highly tumorigenic when explanted into immuno-compromised mice. This effect was highly reproducible, arguing against the acquisition of a unique genetic background predisposing a cell line to become transformed, as the same phenotypes were observed in fibroblasts derived from a total of four individual oncopigs. Taken together, these data support the conclusion that the derived oncopig fibroblasts are inducibly tumorigenic.

Consistent with the ability of the derived oncopig fibroblasts to be tumorigenic, an intramuscular injection of AdCre into transgenic oncopigs resulted in the development of a mesenchymal tumor suggestive of leiomyosarcoma at the site of injection. Again, this result was highly reproducible, being observed not only in a different site within the same pig, but also in different littermate oncopigs. As leiomyosarcoma are of smooth muscle origin, presumably these tumors arose from infection of this muscle type, perhaps in the vasculature or the piloerector muscles. AdCre injection into the testes gave rise to a differentiated tumor likely of sex cord stromal origin. In addition, neither the control non-transgenic pigs injected with AdCre nor the control oncopigs injected with AdGFP developed any tumor mass or pathological changes. Taken together, these data support the conclusion that activation of cells in vivo by AdCre induces tumorigenesis. Moreover, these data also suggest that induction of recombination in other tissues, *vis-à-vis* delivery of AdCre by different means than undertaken here or, in the future by tissue-restricted expression of a Cre transgene, may lead to a host of other cancers. As such, this ‘oncopig’ line provides a genetically malleable model for potentially a wide spectrum of cancers, which should prove invaluable to studies previously hampered by the lack of a large animal model of cancer.

## Materials and Methods

All animal work was conducted according to relevant national and international guidelines. All animal studies and procedures were approved by The University of Illinois Institutional Animal Care and Use Committee (IACUC; Protocol numbers 11221 for pig and 12170 for mouse studies).

### Cloning and sequencing of porcine *KRAS* and *TP53* genes in TOPO shuttle vector

TJ Tabasco porcine bone marrow cells were isolated and frozen at -80°C. Total RNA was extracted from these cells with RNeasy mini kit (QIAGEN, CA), 1ug of which was reverse transcribed into cDNA with the QuantiTect Reverse Transcription Kit (QIAGEN, CA). Ensembl genome browser was used to design the PCR primer sequences for amplification of porcine-specific *KRAS* (ENSSSCG00000000561) and *TP53* genes (ENSSSCT00000019534). The forward and reverse primer sequences for *KRAS* were 5’-CTGCTGAAAATGACTGAATATAAACTT-3’ and 5’-TTACATAATTATACACTTTGTCTTTGA-3’, respectively. The PCR thermal cycling conditions for *KRAS* amplification were 94°C for 10 min, followed by 30 cycles of 94°C for 30 sec, 55°C for 3 min, and 65°C for 1 min with a final 72°C step for 10 min. The forward and reverse primer sequences used for *TP53* were 5’-TGCAATGGCGGAGTCGCAG-3’ and 5’-TCAGTCTGAGTCAGGTCCTTC-3’, respectively. The PCR thermal cycling conditions were 94°C for 10 min, followed by 30 cycles of 94°C for 30 sec, 55°C for 30 sec, and 68°C for 1 min with a final 72°C step for 10 min. *ACTB* was used as an endogenous control. The forward and reverse primer sequences used were 5’-GACATCCGCAAGGACCTCTA-3’ and 5’-ACATCTGCTGGAAGGTGGAC-3’, respectively. The PCR conditions used were the same as for *TP53*. PCR amplified *KRAS* and *TP53* cDNAs were then cloned into pCR2.1-TOPO vectors using the TOPO TA cloning kit according to the manufacturer’s instructions (Invitrogen, CA) and the cDNAs confirmed to have 100% nucleotide identity to porcine *KRAS* and *TP53* (http://useast.ensembl.org/index.html).

### Site-directed mutagenesis of *KRAS* and *TP53* cDNA

QuikChange Site-Directed Mutagenesis Kit (Stratagene, CA) was used to introduce changes to the nucleotide sequence corresponding to the G12D mutation into the cloned porcine *KRAS* cDNA and the R167H mutation into the cloned porcine *TP53* cDNA. The primers used to generate the G12D mutation in *KRAS* were 5’-TGGTAGTTGGAGCTGATGGCGTAGGCAAGAG-3’ and 5’-CTCTTGCCTACGCCATCAGCTCCAACTACCA-3’. The primers used to generate the R167H mutation in *TP53* were 5’-GAGGTGGTGAGGCACTGTCCCCACCAT-3’ and 5’-ATGGTGGGGACAGTGCCTCACCACCTC-3’. Mutations were confirmed by sequencing.

### Cloning of *KRAS* ^*G12D*^ and *TP53* ^*R167H*^ cDNAs into the pIRES vector

The aforementioned *KRAS* ^*G12D*^ cDNA was PCR amplified with primers 5’-CTAGCTAGCTAGCTGCTGAAAATGACTGAATAT-3’ and 5’-CCGCTCGAGCGGTTACATAATTATACAC-3’ for 30 cycles of 94°C for 1 min, 94°C for 30 sec, 60°C for 1 min, and 68°C for 1 min, and the resultant fragment cloned into the NheI and XhoI sites of pIRES (Clontech, CA). The aforementioned *TP53* ^*R167H*^ cDNA was PCR amplified with primers 5’-ACGCGTGGACGTCTTGGCCATATGCAATGGAGGA3’ and 5’-ATAAGAATGCGGCCGCTAAACTATTCAGTCTGAGTCAGGTCC-3’ for 30 cycles of 94°C for 1 min, 94°C for 30 sec, 65°C for 1 min, and 68°C for 1 min, and the resultant fragment cloned into the *Sal*I and *Not*I sites of pIRES containing the *KRAS* ^*G12D*^ cDNA. cDNA sequences of *KRAS* ^*G12D*^ and *TP53* ^*R167H*^ were confirmed correct in the resultant vector by sequencing.

### Cloning CAG promoter into a loxP-STOP(polyA)-loxP containing vector

The pkw15 plasmid containing the CAG promoter and the pkw13 plasmid containing LoxP-STOP(polyA)-loxP were used for the vector construction. A CAG promoter was isolated by SpeI/MfeI digestion and cloned into the pkw13 plasmid at SpeI/EcoRI cloning sites.

### Construction of the inducible *KRAS* ^*G12D*^ and *TP53* ^*R167H*^ oncopig expression vector

The *KRAS* ^*G12D*^- *TP53* ^*R167H*^ –pIRES vector was digested with PvuI and NheI, and the resulting *KRAS* ^*G12D*^-IRES- *TP53* ^*R167H*^-polyA fragments were gel purified and cloned into CAG-LSL-pkw13 vectors at PacI/NheI sites. cDNA sequences of *KRAS* ^*G12D*^ and *TP53* ^*R167H*^ in the final vector were confirmed correct in the resultant vector by sequencing.

### Generation of fetal fibroblast strains

Male fetal fibroblasts cells (FFCs) from Minnesota miniature pigs (NSRRC:0005) were collected as described [25] with some modifications. Briefly, after removing the head and internal organs, the fetus was minced and digested individually in 20 ml of digestion media (Dulbecco’s Modified Eagle Medium (DMEM) supplemented with 15% (v/v) fetal bovine serum (FBS), 200 units/ml collagenase and 25 units/ml DNaseI) for 4–5 hrs at 38.5°C and 5% CO_2_ in air. Digested cells were washed with DMEM supplemented with 15% FBS (Hyclone, Logan, UT) and 10 μg/ml gentamicin, cultured overnight, and then collected and frozen at −80°C in FBS supplemented with 10% dimethyl sulfoxide (DMSO) (v/v) and stored in liquid nitrogen.

### Production of transgenic cells

Early passage number FFCs (P1-2) were cultured in cell culture medium (DMEM supplemented with 15% (v/v) FBS, 2.5 ng/ml basic fibroblast growth factor and 10 μg/ml gentamicin) overnight and grown to 75–85% confluency. Media was replaced 4 hrs prior to transfection. FFCs were washed for 1–2 min with phosphate buffered saline (PBS; Invitrogen) and harvested with 0.05% trypsin-EDTA (Invitrogen; 1 ml per 75cm^2^ flask). Cells were resuspended in cell culture medium, pelleted at 600 × g for 10 min, resuspended in 10 ml Opti-MEM (Invitrogen), and then quantified using a hemocytometer and repelleted. Cells were resuspended in transfection media (75% cytosalts [120 mMKCl, 0.15 mM CaCl_2_, 10 mM K_2_HPO4; pH 7.6, 5 mM MgCl_2_] [26] and 25% Opti-MEM [Gibco BRL, Grand Island, NY]). The cell concentration was adjusted to 1×10^6^ cells/ml and 200 μl of cells were co-transfected by electroporation with linearized mutant *KRAS* and *TP53* construct containing a Neo selectable casette (2 μg). Electroporation utilized three consecutive 250-V, 1-ms square wave pulses administered through a BTX ECM 2001 (BTX, San Diego, CA) in a 2 mm gap cuvette. After electroporation, cells were plated in a 100 mm dish at 3,000 cells per dish in cell culture medium. After 36 hrs, cells were selected by the addition of geneticin (G418; 400 μg/ml) for 10–14 days until the formation of cell colonies. Genomic DNA from the cell colonies was used to verify the presence of both transgenes by PCR. These cells then were stored in liquid nitrogen until used as donor cells for SCNT.

### Oocyte maturation, SCNT, and embryo reconstruction

Fibroblast cells identified to have integration of the transgenes (*KRAS* and *TP53*) were used as donor cells for SCNT into enucleated oocytes followed by electrical fusion and activation as previously described [27]. In brief, cumulus-oocyte cell complexes (COCs) were received in Phase I maturation medium from ART Inc. (Madison, WI) approximately 24 hrs after harvest. COCs were then cultured in fresh Phase II maturation medium for a total of 40 hrs in a humidified atmosphere of 5% CO_2_ at 38.5°C. Phase I and II medium were supplied by ART Inc. Expanded COCs were then vortexed in 0.1% hyaluronidase in Hepes-buffered Tyrode’s medium containing 0.01% PVA for 4 min to remove the cumulus cells. Oocytes having a visible first polar body (PB) with uniform cytoplasm were selected and placed in fresh manipulation medium (25 mM Hepes-buffered TCM199 with 3 mg/ml BSA) containing 7.5μg/ml cytochalasin B which was overlaid with warm mineral oil. The PB, MII chromosomes, and a small amount of surrounding cytoplasm of the oocyte were enucleated using a beveled glass pipette with an inner diameter of 17–20 μm. After enucleation, a donor cell was injected into the perivitelline space and placed adjacent to the recipient cytoplasm. Karyoplast–cytoplast complexes were fused and activated with 2 DC pulses (1 sec interval) of 1.2 kV/cm for 30 μsec provided by a BTX Electro-cell Manipulator 200 in fusion medium (0.3 M mannitol, 1.0 mM CaCl_2_, 0.1 mM MgCl_2_, and 0.5 mM Hepes, pH adjusted to 7.0–7.4). After simultaneous fusion and activation, only the fused embryos were cultured into four well cell plates (Nunc, Denmark) containing 500μl of PZM3 with 0.3% BSA and 500nM Scriptaid at 38.5°C and 5% CO_2_ in humidified air for 14 to 16 hrs, until embryo transfer [27].

### Embryo transfer and piglets production

More than 100 SCNT zygotes were surgically transferred to the oviducts of surrogates on the day of, or one day after, the onset of estrus. The pregnant surrogates were monitored via ultrasound throughout pregnancy. Piglets were delivered via cesarean section from surrogates by day 114–116 of gestation. Piglets are processed immediately and tissue samples were collected for establishment of cell lines and PCR genotyping. Piglets were then hand-raised until weaning (3–4 wks of age).

### Generation and treatment of transgenic cells

Each cloned piglet was ear notched for identification and this tissue was used to establish *in vitro* cell cultures. The skin biopsies were, minced and incubated overnight in collagenase type II (400 U/ml, Life, USA), dissolved in DMEM (Life, USA) supplemented with 20% heat-inactivated fetal bovine serum (FBS, Hyclone, Logan, UT, USA), 1% antibiotics/antimycotic solution (penicillin/streptomycin, Life, USA) at 37°C and 5% CO_2_ in air. Twenty-four hours later, cells were dislodged from digested tissue by repeated pipetting and were passed through 100 μm sterile netting into sterile 50ml centrifuge tubes. The samples were centrifuged for 5 min at 1200 rpm, and the cell pellet was resuspended in DMEM, 20% FBS, 1% penicillin/streptomycin to be plated in a 25 cm^2^ tissue flask at 37°C/5% CO_2_. Following 10 passages using limiting dilution, cell lines from each founder animal were characterized as mesenchymal origin (vimentin positive) fibroblast-like cell lines and were frozen for use as a source of cells for experiments. For activation, cells were grown to 80% confluency, medium changed to 5% FBS and AdCre or AdGFP (Vector Biolabs, PA) was added at 200 to 400 MOI. Cells were incubated for 5 hrs at 37°C then replenished with fresh medium. AdCre and AdGFP cell lines were maintained separately and each of the mesenchymal origin (vimentin positive), fibroblast-like cell lines were used for assays and histological analyses. These fibroblast-like cell lines were passaged to senescence (pass 45 or less for non-transgenic and AdGFP treated transgenic cells) and the AdCre treated transgenic lines have been passaged to 95 passes or greater.

### Genotyping assay

DNA and RNA were isolated from cultured cells using the AllPrep DNA/RNA Mini Kit (Qiagen, USA). Total RNA (1 μg) was reverse transcribed into cDNA in a 20 μl reaction mixture using an Omniscript RT kit (Qiagen, USA) and 1 μl was used in a 25 μl PCR mixture of HotStarTaq Plus DNA Polymerase kit (Qiagen, USA). Primers used for amplification of *TP53* were 5’-TGGCTCTCCTCAAGCGTATT-3’ and 5’-ATTTTCATCCAGCCAGTTCG-3’. Primers used for amplification of *KRAS* were 5’-TTGTACAGCTAGCTGCTGAAAATGACTGAATAT-3’ and 5’-ATTCTCGAGCGGTTACATAATTATACAC-3’. Primers used for amplification of *CAG* were 5’-TCATATGCCAAGTACGCCCC-3’ and 5’-CCCCATCGCTGCACAAAATA-3’. PCR amplification was performed by 30 cycles of 94°C for 1 min, 94°C for 30 sec, 60°C for 1 min (*KRAS* ^*G12D*^ and CAG) or 58°C for 1 min (*TP53* ^*R167H*^), followed by a final incubation of 72°C for 10 min. The same primers and conditions described above were used for the *ACTB* control.

### Cell proliferation assay

Following the Cell Trace CFSE Cell Proliferatoin Kit (Molecular Probes) protocol, each cell line was trypsinized, washed, and resuspended in single cell suspension at 1×10^6^ cells per ml in PBS, 0.1% BSA. The CFSE dye (CAS number 150347-59-4) was added to a final working concentration of 10 μM. The cells were incubated for 10 min at 37°C followed by quenching with 5 volumes of ice old culture media pelleting and then three washes with fresh media. An aliquot of cells were analyzed by flow cytometry (BD LrsII) with 488 nm excitation and fluorescein emission filters. The remaining cells were plated in wells. At each time point of analysis, cells were trypsinized, washed, and resuspended for flow cytometry analysis. Average Mean fluorescent units (MFU) were recorded and analyzed.

### Cell migration analysis

The ability of cells to migrate in monolayer cultures was assessed by a scratch wound assay [28]. The Ibidi Culture-Insert (Ibidi, Verona, WI) was used with confluent cell cultures. The distance and quantity of cell migration into the cell-free zone was evaluated on a digital camera attached to an inverted microscope at 0, 12 and 24 hrs (Carl Zeiss Microscopy, LLC, United States). The recorded images were analyzed using AxioVision Microscope software (Carl Zeiss Microscopy, LLC, United States). The data were expressed as the mean ± SEM and the experiment was run in triplicate.

### Growth in soft agar analysis

Growth of fibroblast cells treated with AdGFP or AdCre in soft agar was performed as previously described [6] with minor modifications. Specifically, 2×10^4^ cells were in DMEM plus 10% calf serum in 0.33% (w/v) noble agar were plated above 0.5% noble agar. Cells were fed weekly by the addition of DMEM (200 μl) supplemented with 10% calf serum. After 2 wks colonies were scored by counting under a microscope and AxioVision software was used to count the colonies.

### Xenograft tumorigenesis assay

Under an approved protocol by the University of Illinois Institutional Animal Care and Use Committees (IACUC), 5x10^6^ cells were mixed with Matrigel (BD Biosciences, San Diego, CA, USA) and injected subcutaneously into the flanks of 3 to 4 severe combined immunodeficient female mice (NOD.CB17-Prkdcscid/ JAX, Bar Harbor, ME) per cell line. Each mouse was injected with the same cell line, the control AdGFP treated cells into the left flank and the AdCre treated cells into the right flank. Calipers were used to measure the tumors approximately 3 times per week. Tumor volumes were calculated using the equation 1/2 length^2^ x width in the unit of mm^3^. At the termination of the growth study all animals were euthanized and tumor tissue collected for molecular analysis and histopathology.

### AdCre induction of tumors in pigs

All animal studies and procedures were approved by the University of Illinois IACUC. Transgenic clone 63–3 was selected as the sire line for producing pigs for further in vivo studies. Clone 63–3 was chosen based on fertility as well as having a single transgene integration site on SSC18. Three 5-week old transgenic piglet offspring resulting by breeding clone 63–3 with a domestic dam were anesthetized (TKX, 1 ml/50 lbs) and injected. A volume of 0.5 or 1.0 ml of AdCre or AdGFP precipitate was injected subcutaneously (SQ), intratesticularly (IT) or intramuscularly (IM) using a 21 gauge needle. The precipitate was made as previously described [29]. In short, Ad5CMVCre-eGFP (AdCre) or Ad5CMV-eGFP (AdGFP, Gene Transfer Vector Core, University of Iowa) was diluted with minimal essential medium (MEM, GIBCO) to a final concentration of 2×10^9^ PFU/ml and 2 M Calcium chloride (to 0.01M final concentration) was added, mixed, and allowed to incubate at room temperature for 15 min prior to injection. All injections were completed before 45 min of incubation and were monitored daily and ultrasound imaging done on day 10 post injection.

### Immunocytochemistry (IHC)

Histological sections of tumor tissues were analyzed following H&E staining and additional immunostaining on selected sections to determine cell origins. IHC was performed on 4 μm slides of formalin-fixed and paraffin-embedded tumor tissue specimens. The sections were deparaffinized with changes of xylenes and graded ethanols to water. Antigen retrieval was performed in a Decloaking Chamber (Biocare) with Diva DeCloaker solution (Biocare Medical, DV 2004). Staining procedures were performed on a Biocare IntelliPath autostainer. Blocking was performed using Peroxidazed 1 (PX968, Biocare Medical) for 5 min, and Background Punisher (BP974, Biocare Medical) for 10 min. Sections were then incubated for 30 min at room temperature with a prediluted monoclonal antibody (Vimentin, SM Actin, MS Actin or Cytokeratin; Biocare Medical). Secondary antibody, mouse on canine polymer (Biocare Medical, MC541) was applied for 30 min. Finally, the chromagen, diaminobenzidine (DAB) was incubated for 5 min (IP FLX DAB, IPK5010, Biocare Medical) and followed by hematoxylin counterstain (CATHE, Biocare Medical) for 2 min. Negative controls were obtained by replacing the primary antibody with Polymer negative control serum (Biocare Medical, NC499) for 30 min.

### RNA isolation

Total RNA was extracted from frozen tissue samples using the AllPrep DNA/RNA Mini Kit (Qiagen, Valencia, CA, USA) following the manufacturer’s protocol. RNA concentrations were determined using a NanoDrop spectrophotometer and analyzed by an Agilent 2100 Bioanalyzer using an RNA Nano bioanalyzer chip to determine RNA integrity as well as the presence/absence of gDNA by the Carver High-Throughput DNA Sequencing and Genotyping Unit (HTS lab, University of Illinois, Urbana, IL, USA). Only RNA samples with a RNA integrity number (RIN) greater than 7 were used for sequencing.

### RNA-seq library preparation

High-quality RNA (1**μ**g) was used to generate TruSeq Stranded RNA-seq libraries (TruSeq Stranded RNA Sample Preparation Kit, Illumina, San Diego, CA, USA) following standard protocols. Briefly, messenger RNA was isolated from the high quality DNAse treated total RNA and first-strand synthesis performed with a random hexamer and SuperScript II (Life Technologies, Carlsbad, CA, USA). Second-strand synthesis was performed using dUTP instead of dTTP. Double stranded DNA was blunt-ended, 3’-end A-tailed and ligated to indexed adaptors. The adaptor–ligated double-stranded cDNA was amplified by PCR for 10 cycles with the Kapa HiFi polymerase (Kapa Biosystems, Woburn, MA, USA) to reduce the likeliness of multiple identical reads due to preferential amplification. The final libraries were quantified using Qubit (Life Technologies, Carlsbad, CA, USA) and the average size was determined on an Agilent bioanalyzer DNA7500 DNA chip (Agilent Technologies, Wilmington, DE, USA) and diluted to 10 nM. The 10 nM dilution was further quantitated by qPCR on an ABI 1900 to ensure high accuracy quantification for consistent pooling of barcoded libraries and maximization of the number of clusters in the Illumina flowcell.

### RNA-seq sequencing

RNA-seq Illumina sequencing was performed on libraries multiplexed and loaded onto 8-lane flowcells for cluster formation and sequenced on an Illumina HiSeq2000. The libraries were sequenced to a total read length of 100 bp from both ends (paired-end sequencing) of the molecules. The run generated. bcl files which were converted into demultiplexed compressed fastq files using Casava 1.8.2 (Illumina, San Diego, CA, USA).

### RNA-Seq data analysis

An average of 70 million raw stranded paired-end reads were produced for each sample, ranging from 54.6 to 84.7 million. Raw reads were trimmed sequentially for adapter contamination, A-tails, and minimum quality score (20) and minimum length (20 bp) using Trim Galore v.0.3.3 (http://www.bioinformatics.babraham.ac.uk/projects/trim_galore/). Unpaired reads were retained with a minimum length of 35 bp. Trimmed paired and unpaired reads were aligned to the swine reference genome using Tophat v.2.2.10 [30]. Tophat analysis included a pre-alignment to the reference genome to filter out reads extending the maximum number of alignments (-M option) followed by alignment to the Ensembl swine reference transcriptome (-G) and alignment to the genome. The number of allowed alignment hits (-g option) was 20. Furthermore the–read-realign-edit-dist option was set to 0, the–mate-inner-dist option to 120, the–mate-std-dev option to 260 and included the fr-firststrand option. Aligned bam files were assessed for differential gene expression using cufflinks v.2.2.1 [31]. First cufflinks was used to assemble transcripts for each sample using the fr-firststrand option, followed by Cuffmerge to merge the assembled transcripts from all samples with the reference transcripts. Cuffquant was used to pre-compute gene expression levels for each sample using the–u option, which more accurately weights reads mapping to multiple locations, and the fr-firststrand option. Finally, Cuffnorm was used to produce gene expression levels normalized for library size by setting the–library-norm-method to geometric and including the fr-firststrand option. Samtools mpileup [32,33] was used to identify the ratio of WT and mutant TP53 and KRAS gene expression based on the reads overlapping the mutation site. RNA-seq data are available in the ArrayExpress database (www.ebi.ac.uk/arrayexpress) under accession number E-MTAB-3382.

### Identification of transgene insertion site

Genomic DNA was isolated from the 63–3 cell line and used for genome walking (Universal Genome Walker Kit; Clontech, Mountain View, CA). Briefly, four pools of adaptor-ligated genomic DNA were produced by restriction digestion and subsequent ligation with a single universal adaptor according to the manufacturer’s recommendation. Two sets of nested oligonucleotide primers were designed corresponding to opposite ends of the transgene construct and were oriented for the amplification of flanking genomic DNA (i.e., primers were designed from the minus strand for the 5’ end of the construct and plus strand for the 3’ end of the construct). An initial PCR amplification was performed using the innermost primers corresponding to each of the construct ends and a primer directed at universal adapter sequence. First round amplicons were used as template for a second round of amplification with the appropriate nested primer sets. PCR products were separated on a 1.2% agarose gel; bands of interest were extracted and purified using NucleoSpin Gel and PCR Clean-up kit (Clontech) and then sequenced. Genomic sequences were mapped to the pig genome using BLAST [34]
